# Response of Soil Nematode Community Structure and Function to Monocultures of Pumpkin and Melon

**DOI:** 10.3390/life12010102

**Published:** 2022-01-12

**Authors:** Dan Zhao, Yao Wang, Ling Wen, Hongyun Qu, Zuobiao Zhang, Hui Zhang, Yunhe Jia, Juan Wang, Yixin Feng, Yan Li, Fan Yang, Fengjuan Pan

**Affiliations:** 1 Key Laboratory of Mollisols Agroecology, Northeast Institute of Geography and Agroecology, Chinese Academy of Sciences, Harbin 150081, China; 2191589@s.hlju.edu.cn; 2 Horticultural Branch, Heilongjiang Academy of Agricultural Sciences, Harbin 154026, China; zd1978722@163.com (D.Z.); wenling578@163.com (L.W.); qzqx2002@163.com (H.Q.); zhangzuobiao@163.com (Z.Z.); la_jiao800@163.com (H.Z.); 800530jyh@163.com (Y.J.); wangjuan.lc@163.com (J.W.); fyx0451@163.com (Y.F.); luckyone_yan@yahoo.com (Y.L.); yangfan19830624@163.com (F.Y.)

**Keywords:** soil nematodes, monoculture, community structure, soil food web

## Abstract

It is well known that crop monoculture can induce negative effects on soil ecosystems and crop productivity. However, little is known about how vegetable monoculture affects the soil nematode community structure and its relationship with vegetable yields. In this study, the composition, abundance, metabolic footprint, and ecological indices of soil nematodes are investigated in monocultures of pumpkin and melon. The relationships between nematode community structure and yields of pumpkin and melon were analyzed by linear regression. Both monoculture soils of pumpkin and melon suppressed the relative abundance of bacterivores but increased the relative abundance of plant parasites. Pumpkin monoculture soils decreased soil nematode diversity but increased the maturity index of plant parasites. Monoculture soils of pumpkin and melon decreased the metabolic footprint of lower- and higher-level trophic groups of the soil food web, respectively. Pumpkin and melon monoculture soils increased the food web indices channel index (CI) but decreased the enrichment index (EI) and the structure index (SI). The monoculture soils of pumpkin and melon led to a more fungal-dominated decomposition pathway and degraded soil food web conditions. The abundance of bacterivores and food web indices EI and SI were positively correlated with soil nutrients and pH, while the abundance of plant parasites and CI were negatively correlated with soil nutrients and pH. *Paratylenchus* was negatively correlated with pumpkin and melon yields and could be the potential plant parasites threatening pumpkin and melon productions. Redundancy analysis showed that monocultures of pumpkin and melon altered the soil nematode community via soil properties; total N, total P, alkeline-N, and pH were the main driving factors.

## 1. Introduction

Monoculture is a very common practice in the intensive production of horticultural vegetables for profit-driving. In monoculture conditions, plant roots secrete the same exudates and provide the same straw degradant to the soil, resulting in changes in the soil physicochemical properties, microbial biomass and activity, and composition and function of soil biota and deeper changes in soil ecosystem function [1,2]. Harmful organisms are thought to build up over continuous monoculture, and this results in monoculture yield decline [3,4]. Therefore, previous studies on soil organisms in monoculture soils have mainly focused on pathogens [1]. Soil organisms are essential for maintaining soil health, which is one of the most important requirements for plant production in agricultural systems [5]. Recently, increasing numbers of studies have reported that monoculture has led to the disruption of soil microbial community structure [2,6]. However, less is known about the impact of vegetable monoculture on soil nematode diversity.

Soil nematodes are important belowground organisms in agroecosystems [7]. Many studies have shown that soil nematodes participate in various soil processes [8,9,10]. Free-living nematodes are considered beneficial organisms in soil, promoting soil N mineralization and phosphorus mineralization and increasing soil enzyme content by regulating bacterial community structure [11,12,13]. Plant-parasitic nematodes are harmful organisms and are estimated to cause about USD 173 billion in global yield loss each year [14]. Some plant-parasitic nematode species such as *Heterodera glycine*, *Ditylenchus destructor,* and *Meloidogyne luci* have been reported to seriously affect agricultural production, and the latter two species have developed new hosts [15,16]. Soil nematode abundance and food web indices provide useful information about the soil food web’s ecological structure and functions [17,18,19]. Biological assessment is necessary for understanding the deep changes in the soil ecosystem induced by different agricultural management. Soil nematodes have been widely used as bioindicators to assess soil conditions in different soil environments impacted by land use, organic amendments, and animal grazing [20,21,22].

Vegetables are one of the most vital foods for human sustenance and have the largest cultivation area apart from agricultural crops, with their cultivation area having reached 21,289,000 hm^2^ in China [23]. Previous studies have been more focused on the influences plant-parasitic nematodes on important vegetables such as *Meloidogyne* and *Ditylenchus*, which have caused serious damage to tomato, cucumber, and sweet potato [24,25,26]. Some studies reported that the influences of organic manure and chemical fertilizer on soil nematode community structure in vegetable soils [27,28,29]. However, little is known about the effect of monoculture vegetables on soil nematode fauna. The decrease in the yield of monoculture plants is mainly attributed to autotoxicity and the accumulation of pathogens and other pests [30,31]. It is unclear whether the yield of monoculture plants is related to soil nematode diversity and trophic groups besides plant parasites.

Pumpkin and melon, cucurbitaceous vegetables, have been widely cropped in tropical and subtropical regions. Both vegetables have high nutritional and economic values. In this study, soil nematode community structure, metabolic footprints, soil properties, and vegetable yield are investigated in monoculture fields of pumpkin and melon. The objectives of the study are to determine (1) the influence of pumpkin and melon monoculture on soil nematode community structure and metabolic footprints, (2) the relationship between soil nematode community structure and yields of pumpkin and melon in monoculture soils, and (3) potentially harmful plant parasites in monoculture soils of pumpkin and melon. This study provides valuable information on the influence of vegetable monoculture on soil biodiversity and function.

## 2. Materials and Methods

### 2.1. Experimental Design

This study was conducted in fields protected by a polypropylene greenhouse. The protected fields are located at the Horticultural Branch, Heilongjiang Academy of Agricultural Sciences, Heilongjiang province, China (45.635525° N, 126.652464° E). The region has a continental monsoon climate with annual precipitation of 400–600 mm, approximately 65% of which occurs from June to August. The mean monthly air temperature varies from −23 °C in January to 21 °C in July. The soil is a typical black soil, with 20.1 g kg^−1^ of total organic matter, 1.6 g kg^−1^ total N, and a pH of 6.6 at 0–20 cm depths.

The experiment was based on pumpkin and melon breeding in protected fields that were planted with pumpkin and melon for one year and four years. The treatments included the following: (1) 4-year pumpkin monoculture (*Cucurbita* *maxima*, Longyuanlixiang, PM), (2) 4-year melon monoculture (*Cucumis melo* L., Longtian 6, MM), (3) 1-year pumpkin cropping (*Cucurbita* *maxima*, Longyuanlixiang, PR), and (4) 1-year melon cropping (*Cucumis melo* L., Longtian 6, MR). Each treatment had three replicates of 25-m^2^. Eggplant, tomato, and pepper were alternatively planted in this protected field before pumpkin and melon. Hymexazol was applied in the soil to suppress the population of fungal pathogens in spring. Chicken manure compost was used as a base fertilizer and applied before ridging in spring. The manure used was a chicken manure compost (50 t ha^−1^) with 41.1% water content, containing 401.5 g organic matter kg^−1^, 16.3 g N kg^−1^, 15.4 g P_2_O_5_ kg^−1^, and 8.5 g K_2_O kg^−1^ on a dry weight basis. The chicken manure was applied before ridging. Vegetable manure compound fertilizer (1.2 t ha^−1^), containing 200 g organic matter kg^−1^, 80 g N kg^−1^, 80 g P_2_O_5_ kg^−1^, and 80 g K_2_O kg^−1^, was applied with the sowing of pumpkin and melon. The yields of pumpkin and melon were measured at harvest time.

### 2.2. Soil Sampling and Analysis

The plants were transferred to experimental greenhouses after three weeks of seeding, and the required irrigation and fertilizer (leaf fertilizer) were applied during the growing season. After 3 months of the transplanting, soil samples were collected from 0–20 cm depths with a shovel around plants. Six soil samples were collected from each replicate, and all soil samples were sieved and mixed well by hand. Some soils were air-dried for soil property measurement, which was performed using standard methods for all soil samples. The analyzed soil properties included total soil organic carbon (SOC), total N (TN), alkeline-N (AN), soil pH, total P (TP), and Olsen P/available P (AP) [32]. Leftover soils were stored in a refrigerator at 4 °C for determining soil nematode community.

### 2.3. Soil Nematode Extraction and Identification

Nematodes were extracted from 100-g fresh soil for 72 h using the Baermann tray method, modified from the Baermann funnel method [33]. The extracted nematodes were heat-killed (60 °C) and preserved in 50-mL tubes containing 40 g L^−1^ formaldehyde solution. One-quarter of each nematode suspension was observed under a microscope (Olympus BX43, Tokyo, Japan) at 200× or 400× and was identified to the genus level using diagnostic keys. The soil nematodes were assigned to four trophic groups: plant parasites (PP), bacterivores (Ba), fungivores (Fu), and omnivores/predators (OP), with corresponding colonizer–persister (cp) groups [34,35]. The abundance of soil nematodes was adjusted to the number of individuals per 100 g of dry soil. The nematode length (µm) and maximum body diameter (µm) were determined using an ocular micrometer for metabolic function calculation.

### 2.4. Soil Nematode Community and Data Statistic

The ecological indices, including the Shannon–Wiener index (H′), maturity indices of plant-parasitic nematodes (PPI) and free-living nematodes (MI), represent the soil nematode diversity and the life-history characteristics of plant-parasitic nematodes and free-living nematodes [36,37]. The enrichment index (EI), structure index (SI), and channel index (CI) were calculated to estimate the effect of vegetable cultivation on the soil food web [38]. The metabolic footprints of nematodes (F) were computed for each sample based on nematode biomass (W) [18]. These indices were calculated as follows:

H′ = −∑p_i_lnp_i_, where p_i_ is the proportion of individuals in the ith taxon. (1)

PPI = ∑v_i_ f′_i_, where v_i_ is the *c*-*p* value of ith taxon, and f′_i_ is the frequency of ith taxon. (2)

CI = 100 × 0.8 Fu2/(3.2 Ba1 + 0.8 Fu2)(3)

EI = 100 × (*e*/(*e* +*b*)) (4)

SI = 100 × (*s*/(*s* + *b*)) (5)

*e* = ∑(3.2 Ba1 + 0.8 Fu2) (6)

*b* = ∑0.8 (Ba2 + Fu2) (7)

*s* = ∑(1.8 Ba3 + 3.2 Ba4 + 3.2 Ca4 + 5.0 Ca5) (8)

where Ba, Fu, Om, and Ca represent bacterivores, fungivores, omnivores, and predators, respectively; these variables were followed by *c*-*p* values to represent the functional guilds of each trophic group [18].

W = (D2 × L)/(1.6 × 10^6^) (9)

F = ∑ (Nt (0.1 (Wt/mt) + 0.273 (Wt0.75))(10)

where W is the fresh weight (µg), D is the greatest body diameter (µm), L is the nematode length (µm), Nt is the number of t taxa, and mt is the *c*-*p* value.

The metabolic footprints of plant parasites, bacterivores, fungivores, and omnivores/predators were abbreviated as PPF, BaF, FuF, and OPF, respectively. The enrichment footprint (EF) and the structure footprint (SF) were calculated by summing the enrichment component and the structure component; the area composed of the enrichment footprint and the structure footprint is the functional footprint [18].

### 2.5. Data Analysis

An independent-samples *t*-test was performed to assess the effects of pumpkin and melon on abundance, ecological indices, and metabolic footprint of soil nematodes. Pearson analysis was conducted to evaluate the correlation between the abundance, ecological indices, and metabolic footprint of soil nematodes and soil properties. Regression analysis was conducted to evaluate the relationship between abundance and ecological indices of soil nematodes and yields of pumpkin and melon. All of the statistical tests were conducted using SPSS version 16.0 statistical software package (SPSS, Chicago, IL, USA). Differences were considered significant at the *p* < 0.05 level. Non-metric multidimensional scaling (NMDS) based on Bray Curtis distance was applied to analyze composition changes at the genus level. The relationship between the community structure and environmental parameters was analyzed by redundancy analysis (RDA) using CANOCO version 5.0 software.

## 3. Results

### 3.1. Soil Nematode Abundance and Metabolic Footprint

**Figure 1:**
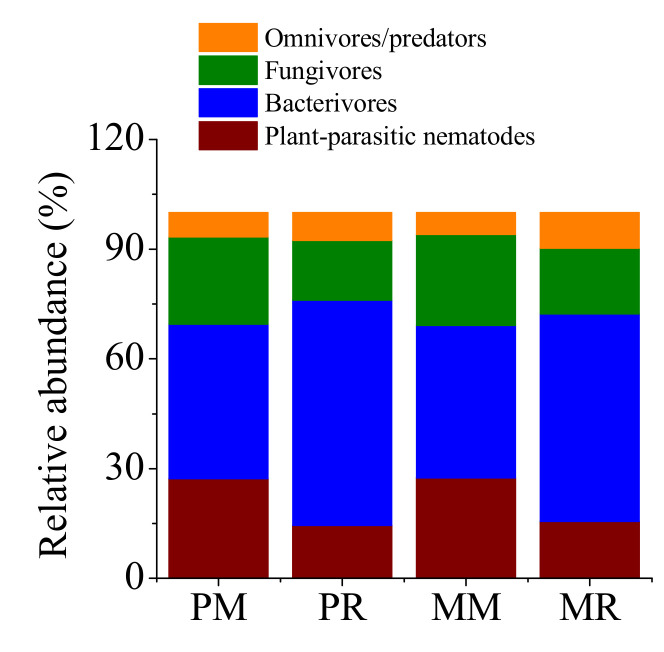
Soil nematode abundance in pumpkin and melon fields. PM, 4-year pumpkin monoculture; PR, 1-year pumpkin cropping; MM, 4-year melon monoculture; MR, 1-year melon cropping. Same color columns with different lower-case letters indicate a significant difference (*p* < 0.05) between monoculture and 1-year cropping soils.

The monoculture soils of pumpkin and melon increased the relative abundance of plant parasites and fungivores but decreased the relative abundance of bacterivores compared with 1-year cropping of pumpkin and melon, respectively (Figure 1). In addition, MM decreased the abundance of omnivores/predators compared with MR.

Pumpkin monoculture influenced the soil nematode metabolic footprint (Table 1). Compared with PR, PM increased the metabolic footprint of plant parasites and fungivores but decreased the metabolic footprint of bacterivores.

### 3.2. Soil Nematode Community Composition

A total of 28 taxa were observed in this study (Table 2). Bacterivores were the most abundant with 11 taxa, followed by plant parasites with 7 taxa, and fungivores and omnivores/predators, each with 5 taxa. The MR had the largest number of taxa, with 25 genera, followed by PR with 24 genera, and PM and MM had the least number of taxa, with 17 genera. Among them, *Paratylenchus* (MM) was the most abundant plant parasite, *Aphelenchus* (PM) was the most abundant fungivore, *Eucephalobus* (PR) was the most abundant bacterivore, and *Microdorylaimus* (PR) was the most abundant omnivore/predator.

**Figure 2:**
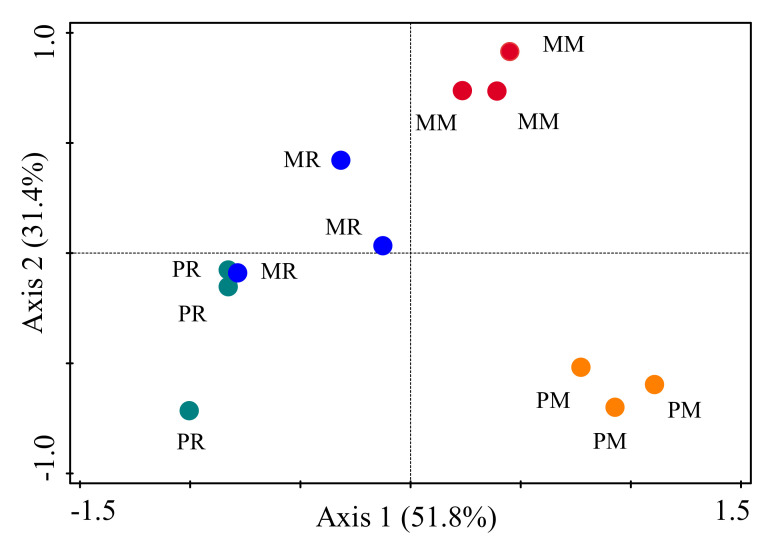
Non-metric multidimensional scaling (NMDS) plots based on genera of soil nematodes among different treatments. PM, 4-year pumpkin monoculture; PR, 1-year pumpkin cropping; MM, 4-year melon monoculture; MR, 1-year melon cropping.

Our non-metric multidimensional scaling analysis showed an obvious shift in community composition in terms of the genus of soil nematodes (Figure 2). In terms of genus composition, three aggregation zones were clearly defined, which represent the cases for PM, MM, and MR plus PR.

### 3.3. Soil Nematode Ecological Indices

**Figure 3:**
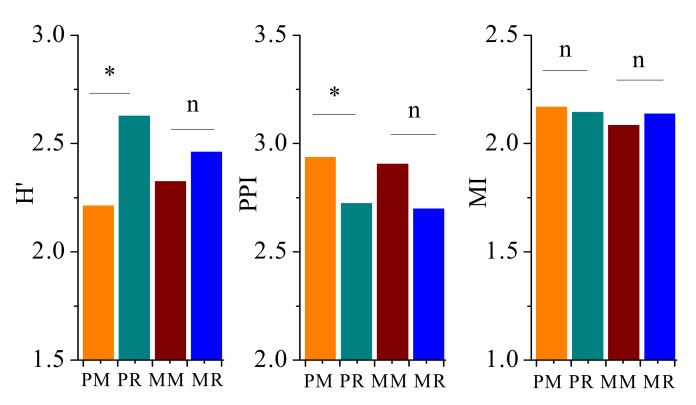
Community indices of soil nematodes in pumpkin and melon fields. H′, Shannon–Wiener index; PPI, maturity index of plant-parasitic nematodes; and MI, maturity index of free-living nematodes. PM, 4-year pumpkin monoculture; PR, 1-year pumpkin cropping; MM, 4-year melon monoculture; MR, 1-year melon cropping. “*” indicates a significant difference at the *p* < 0.05 level; n indicates a non-significant difference.

**Figure 4:**
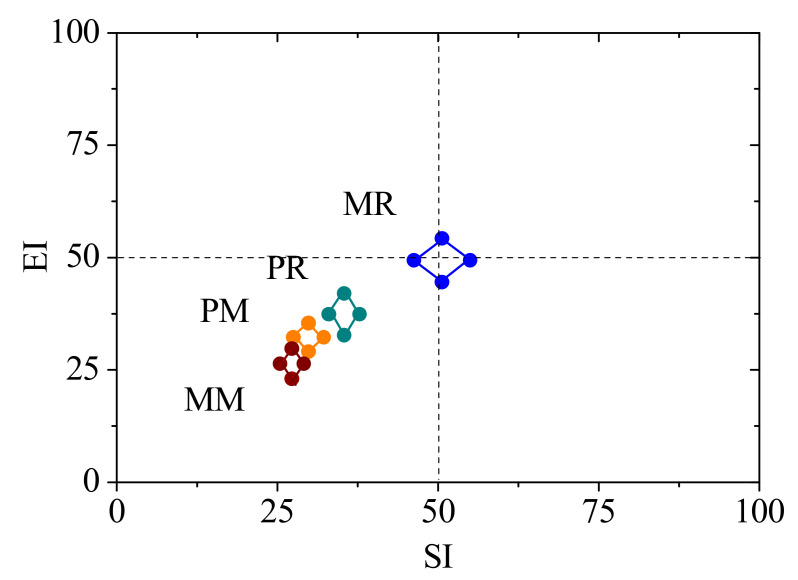
Food web indices and functional metabolic footprint of nematodes subjected to the effects of pumpkin and melon monocultures. PM, 4-year pumpkin monoculture; PR, 1-year pumpkin cropping; MM, 4-year melon monoculture; MR, 1-year melon cropping. EI, enrichment index; SI, structure index. The functional metabolic footprint is described by the sequentially joining points: (SI − 0.5Fs, EI); (SI + 0.5Fs, EI); (SI, EI + 0.5Fe); (SI, EI − 0.5Fe). Fe and Fs represent the enrichment footprint and the structure footprint, respectively. The nematode functional metabolic footprint is the total area of the two functional (enrichment and structure) footprints [18].

**Figure 5:**
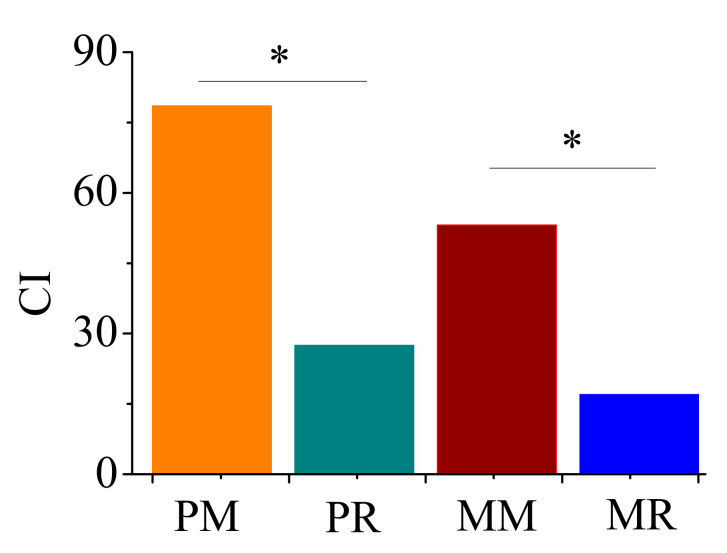
Channel index of soil nematodes in pumpkin and melon fields. PM, 4-year pumpkin monoculture; PR, 1-year pumpkin cropping; MM, 4-year melon monoculture; MR, 1-year melon cropping. “*” indicates a significant difference at the *p* < 0.05 level.

The ecological indices of soil nematodes were evaluated in this study (Figure 3, Figure 4 and Figure 5). There was no significant difference in ecological indices H′, PPI, and MI between MM and MR. The PM decreased the H′ but increased the PPI compared with PR (Figure 3).

Monocultures of pumpkin and melon affected the nematode food web structure (Figure 4 and Figure 5). In the SI vs. EI graph, MR soils were located in the center of the graph, and PM, PR, and MM soils were located in the lower-left quadrant (Figure 4). Both PM and MM increased the CI value compared with PR and MR, respectively (Figure 5).

### 3.4. Correlation of Soil Nematodes with Soil Properties

The monoculture soils of pumpkin and melon decreased the soil nutrients and pH (Table S1). Pearson analysis between soil nematode parameters and soil properties showed a greater positive correlation than a negative correlation (Table 3). Plant parasites, PPI, and CI were negatively correlated with SOC, total N, total P, available P, alkeline-N, and pH. Bacterivores and H′ were positively correlated with total N, total P, alkeline-N, and pH. The indices EI and SI were positively correlated with SOC, total P, alkeline-N, and pH. 

**Figure 6:**
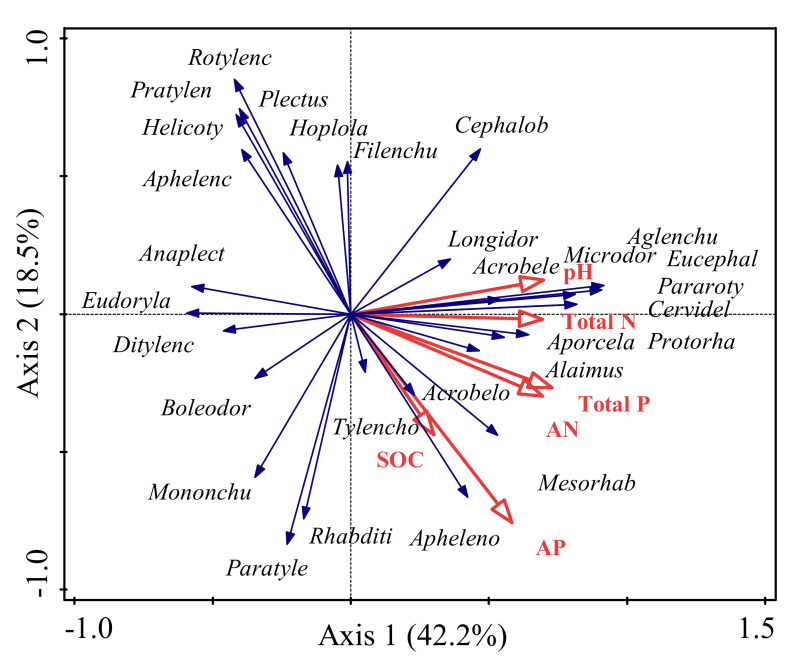
Redundancy analysis (RDA) of the relationship between soil properties and abundance of soil nematode genera. SOC, soil organic carbon; TN, total soil nitrogen; AN, alkaline-N; AP, Olsen P. Full names of nematode genera are shown in Table 1.

The first two RDA axes explained 60.7% of the species-environment relationship based on the nematode genera and soil properties (Figure 6). The first axis was primarily driven by total N, total P, alkeline-N, and pH. The main drivers of the secondary axis were AP and SOC. Bacterivores, such as *Alaimus*, *Protorhabditis*, *Cervidellus*, *Eucephalobus*, *Acrobeloides*, and *Mesorhabditis* were positively correlated with all soil nutrients and pH. Plant parasites *Helicotylenchus*, *Pratylenchus*, and *Rotylenchus,* as well as juveniles of Hoplolaimidae, were negatively correlated with the soil nutrients and pH.

### 3.5. Correlation of Soil Nematodes with Yields of Pumpkin and Melon

**Figure 7:**
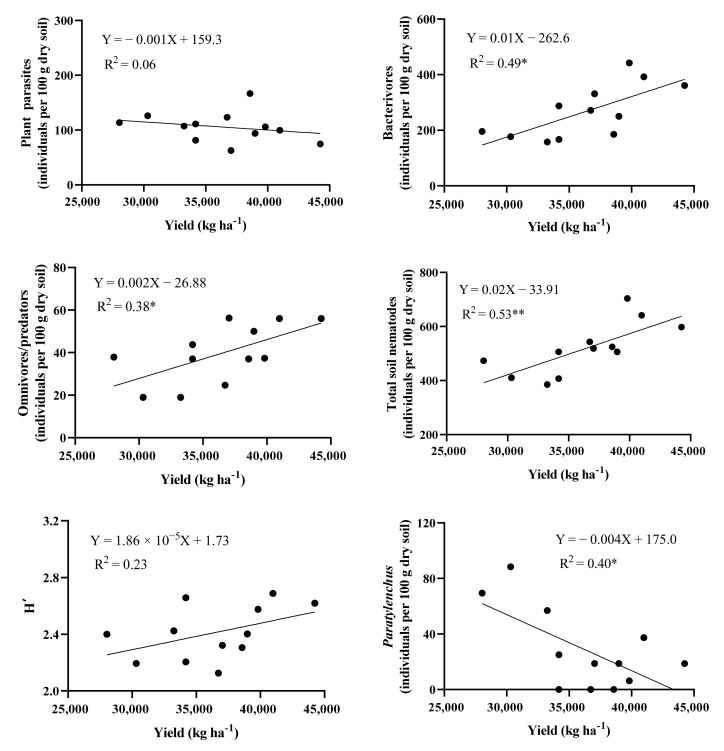
Relationship between soil nematode community and yields of pumpkin and melon. H′, Shannon-Wiener index. “*” indicates significance at *p* < 0.05 level.

The monoculture soils decreased the yields of pumpkin and melon (Figure S1). Linear regression analysis showed that bacterivores, omnivores/predators, and total soil nematodes were positively correlated with the yields of pumpkin and melon (Figure 7). Plant parasite *Paratylenchus* was negatively correlated with the yields of pumpkin and melon. The total abundance of plant parasites and H′ had a non-significant correlation with the yields of pumpkin and melon.

## 4. Discussion

### 4.1. Effects of Vegetable Monoculture on Abundance and Metabolic Footprint of Soil Nematodes

Both monoculture soils of pumpkin and melon had a tendency to reduce the relative abundance of bacterivores but increase the relative abundance of plant parasites (Figure 1). This indicated that monoculture soils of pumpkin and melon decrease the beneficial nematodes and increase the risk of plant disease caused by plant parasites. This is likely due to the accumulation of harmful substances to free-living nematodes or to the decrease of soil microbial abundance in monoculture soils of pumpkin and melon. Previous studies have reported that phenolic acids, which are produced by most plants and are harmful to the plants themselves and most soil organisms, accumulate in continuous cropping soils [39]. Additionally, potato monoculture decreased the abundance of bacteria Acidobacteria and Nitrospirae, which can be the food of bacterivores and omnivores [40]. Our results are consistent with the findings that plant parasites increased after a three-year strawberry monoculture [41] and that the abundance of total soil nematodes and microbivorous nematodes decreased in peanut monoculture soils [42].

Metabolic footprints of soil nematodes can reflect metabolic activity and ecosystem function based on estimation of the carbon utilization in nematode biomass production and respiration [18]. The effect of pumpkin monoculture soils on the metabolic footprint of soil nematodes implied that pumpkin monoculture soils promote the metabolic activity of plant parasites and decrease the metabolic activity of bacterivores and lower-level trophic groups of the food web (Table 1). This is likely because pumpkin monoculture soils increased the abundance of plant parasites as the metabolic footprint of soil nematodes is positively correlated with their abundance based on the equation of the metabolic footprint [18]. The metabolic activity of bacterivores should be directly related to the abundance or activity of soil bacteria since they feed on bacteria. Previous studies have reported that long-term monoculture decreases the enzymatic activity and abundance of soil bacteria [40,43]. This may explain why pumpkin monoculture soils decrease the metabolic activity of bacterivores. The lower trophic group is mainly composed of bacterivores, so the activity of the lower trophic group of nematodes, based on the enrichment footprint, is also decreased in pumpkin monoculture soils. Melon monoculture had almost no effect on the functional footprint of soil nematodes (Figure 4). Considering the different effects of pumpkin and melon monoculture soils on the metabolic footprint of soil nematodes, we also speculate that the effect of vegetable monoculture on the contribution of soil nematode to C utilization varies with plant species.

### 4.2. Soil Nematode Community Structure Modification by Vegetable Monoculture

After monocultures of pumpkin and melon, there was a succession of specifically enriched genera from *Acrobeloides* and *Eucephalobus* in PR to *Acrobeloides* and *Aphelenchus* in PM and from *Acrobeloides* in MR to *Acrobeloides* and *Paratylenchus* in MM (Table 2). Considering the species reduction of soil nematodes at the genus level (Table 2), these results indicated that monoculture soils of pumpkin and melon crops modify the soil nematode community composition. This is likely because different plants secrete different root exudates, and monoculture soils of a single plant result in the accumulation of the same root exudates, forming a particular root microenvironment and unique animal fauna [44]. Genus *Aphelenchus* was the most common fungivorous nematode, and it increased more than *Aphelenchoides* in pumpkin monoculture soils. This indicates that a unique microenvironment formed by pumpkin monoculture facilitates the survival of *Aphelenchus*. A previous study indicated that *Aphelenchus* may have more specialized feeding habits than *Aphelenchoides* [45]. Melon monoculture soils increased the risk of nematode disease for *Paratylenchus*, which is a plant-parasitic nematode, and it became the dominant genus in MM.

NMDS plots showed that PM was clearly separated from MM, and both PM and MM were separated from PR and MR in terms of genus composition (Figure 2). This also suggested that monoculture soils of pumpkin and melon influence the community composition of soil nematodes, while monoculture soils of different vegetables differ in their impacts on the community composition of soil nematodes. This may be due to the accumulation of the same type of root exudates in the monoculture soils of pumpkin/melon. Long-term exposure to this environment can increase the abundance of some organisms and reduce or disappear some organisms and cause changes in soil biological community structure [46]. Most plant-parasitic nematodes are host-specific [20], and bacterivores and fungivores are more dependent on microbial flora [8,47], which would definitely influence the community composition of soil nematodes. The PM was separated from MM based on the genus of soil nematodes. This is likely due to the different plant traits of pumpkin and melon. A previous study has reported that the effect of crop monoculture on soil fungal community structure varied based on crop type [48].

### 4.3. Effects of Vegetable Monoculture on Soil Nematode Ecological Indices

Pumpkin monoculture soils decreased the H′ value (Table 2). This indicated that pumpkin monoculture soils reduce soil biodiversity based on soil nematode indicators. This result is consistent with a previous study showing that strawberry decreased the Shannon–Wiener index of soil nematodes after a three-year monoculture [43]. The PM increased the PPI value (Table 2). This indicated that pumpkin monoculture soils lead to a shift in plant parasites from persisters to colonizers [37]. The PPI is correlated with the survival strategy of plant parasites. For plant parasites, the relative abundance of *Helicotylenchus* and *Rotylenchus* with higher *c*-*p* values increased more in PM than in PR, which probably resulted in a higher PPI value in pumpkin monoculture soils. A previous study has reported that favorable vegetation to plant parasites caused higher PPI values [49]. Considering the melon monoculture soils did not affect the H′, PPI, and MI values in this study, we speculate that the effect of vegetable monoculture on the diversity and maturity of soil nematodes depends on vegetable species.

The CI can indicate the soil organic decomposition pathway or decomposition process. The CI value was increased in pumpkin and melon monoculture soils (Figure 2). This indicated that monoculture soils of pumpkin and melon result in a switch of soil organic matter turnover from a bacterial pathway to a fungal pathway, which is a switch from a quick turnover to a slower turnover of organic matter. According to the faunal analysis profile, RM, PR, and MM soils are located in the lower-left quadrant, which indicates a high level of disturbance, deficiency in soil resource availability, and a stressed food web. The MR soils are located in the center of the graph, suggesting a food web with relatively rich resource availability and complex trophic connections. However, the food web condition found in PR and MR soils was less than that of Li et al., who found a food web with disturbance but rich resource availability in greenhouse vegetable soils with organic amendments [27]. The different results are likely due to the difference in vegetable type and organic matter. Pumpkin and melon were planted with chicken manure compost in our study, but tomato and cucumber were planted with horse manure in the study of Li et al. [27]. Overall, our results imply that monoculture soils of pumpkin and melon modify the food web to a stressed condition, with less available resources and trophic connections.

### 4.4. Factors Controlling the Nematode Community Structure

Plant parasites were negatively correlated with soil properties related to soil nutrients. This result is consistent with previous studies, where plant parasites were inhibited by a fertilizer addition that increased the content of soil ammonium [50]. We also observed a negative relationship between the PPI and soil properties related to soil nutrients. These results indicated that increasing soil nutrients can disturb the community structure of plant parasites and suppress their abundance in vegetable monoculture soils. In addition to the negative effect of ammonium and nitrate related to the content of N on plant parasites, increasing soil nutrients can increase root biomass, produce more harmful exudates for plant parasites, and promote plant defense mechanisms, which may also explain the negative relationship between plant parasites and soil properties. Bacterivores were positively correlated with soil nutrients. This is consistent with previous findings [9] and supports that bacterivores have important contributions to soil nutrient cycling [8]. We found that plant parasites were negatively correlated with soil pH, but bacterivores and omnivores/predators were positively correlated with soil pH. These results are partially inconsistent with previous studies; that is, all nematode trophic groups from the fields of *Brassica rapa* were negatively correlated with pH [51]. These different results are likely due to different soil environments and nematode species, which have different pH tolerance ranges [52,53].

Soil biodiversity is an indicator of soil health, and the H′ was positively correlated with soil nutrients, suggesting that soil nematode diversity could be associated with soil nutrient cycling. There is speculation that the decomposition of wood litter may be more associated with diversity than the abundance of soil epigeic fauna [54], and some studies have proven that soil biodiversity is very important for maintaining soil fertility [55]. The relationship between food web indices (EI, SI, and CI) and soil properties indicates that higher nutrition-rich soil increases the available soil resources of soil nematodes and promotes the structure and trophic links of the soil food web, thereby resulting in a more bacterial-dominated decomposition pathway. This result is in agreement with a previous study that found that the EI and SI were positive but that the CI was negative with increasing soil organic carbon [56]. However, this result is inconsistent with some studies that found that organic matter or a phosphorus/nitrogen addition decreased the SI [10]. This may be due to the different methods, which led to the change in soil properties. The same amount of organic matter was applied in our study, and the changes in soil properties were mainly caused by pumpkin and melon monocultures; however, the difference in soil properties was due to organic matter or fertilizer additions in previous studies [10].

The results of the RDA indicated that the soil nematode community structure was driven by pumpkin and melon monocultures via soil properties (Figure 6). Plant monoculture can cause land degradation and a negative effect on soil chemical properties [57]. For example, plant roots can exude a great variety of compounds, ranging from amino acids, complex polysaccharides, and proteins to smaller volatile lipophilic molecules, into the rhizosphere, all of which can directly or indirectly influence soil properties [58,59]. In this study, pH, total N, alkeline-N, and total P were the main driving factors (Figure 6). It has been proven that the effects of a phosphorus amendment on soil nematodes are powerful in tropical secondary forests [10], and pH is one of the main influence factors regulating the community structure of soil nematodes [60]. Most bacterivores are positively correlated with soil properties, while most plant parasites are negatively correlated. These results are in agreement with the Pearson analysis, further confirming that bacterivores play an important role in soil nutrient cycling.

### 4.5. Correlation of Soil Nematodes with Yields of Pumpkin and Melon

We found that bacterivores and omnivores/predators were significantly positively correlated with the yields of pumpkin and melon (Figure 7). These results indicated that bacterivores and omnivores/predators may be beneficial to vegetable production. Previous studies have reported that free-living nematodes increase mineral N concentration in soil and bacterivores promote the biomass of functional bacteria, such as phosphomonoesterase (ALP)-producing bacteria, which are beneficial to soil ecological function [11,12].

Plant parasites are recognized as one of the greatest threats to crops worldwide [61]. Plant parasite *Paratylenchus* was negatively correlated with the yields of pumpkin and melon (Figure 7). Plant parasites have a stylet that can pierce the plant cell walls to help them get nutrients from the plant. A previous study has shown that *Paratylenchus* is associated with pineapple yield decline [62]. In Belgium, *Paratylenchus* is frequently associated with reduced plant growth of butterhead lettuce monocultured in glasshouses [63]. Our results suggest that *Paratylenchus* could be the potential plant parasites threatening pumpkin and melon production.

## 5. Conclusions

In summary, our findings suggest that vegetable monoculture soils shape the soil nematode community structure. Vegetable monoculture is detrimental to free-living nematodes, and it also increases the relative abundance of plant parasites, which increases the risk of plant disease. Monocultures of pumpkin and melon result in a switch of soil organic matter turnover from a quick turnover that is dominated by a bacterial pathway to a slower turnover that is dominated by a fungal pathway. Pumpkin monoculture soils decreased the carbon utilization of lower trophic groups of the food web. Combined with the effects of pumpkin and melon monocultures on soil nematode community structure, vegetable monoculture degrades the soil biodiversity. The correlations between soil nematode parameters and soil properties imply that increasing soil nutrition can promote the carbon utilization of soil nematodes and the condition of the soil food web. Plant parasite *Paratylenchus* was negatively correlated with the yields of pumpkin and melon, suggesting that *Paratylenchus* could be the potentially plant parasites threatening pumpkin and melon production. Our findings provide a better understanding of the response of soil biodiversity to vegetable monoculture in fertile soil.

## Figures and Tables

**Table 1:** Metabolic footprints of soil nematodes in pumpkin and melon fields. Abbreviations PPF, BaF, FuF, and OpF represent the metabolic footprints of plant parasites, bacterivores, fungivores, and omnivores/predators, respectively. PM, 4-year pumpkin monoculture; PR, 1-year pumpkin cropping; MM, 4-year melon monoculture; MR, 1-year melon cropping. Lines with the same or no lower-case letters are not significantly different (*p* < 0.05) between PM and PR or MM and MR.

Metabolic Footprint	PM	PR	MM	MR
PPF	11.3 ± 2.0 a	3.8 ± 0.5 b	3.5 ± 0.6	4.4 ± 1.8
BaF	30.6 ± 6.7 b	46.9 ± 2.2 a	27.5 ± 2.2	35.7 ± 5.7
FuF	5.9 ± 0.3 a	4.6 ± 0.4 b	5.2 ± 0.3	4.0 ± 0.6
OpF	4.8 ± 1.1	4.4 ± 0.8	3.7 ± 1.8	7.1 ± 0.3

**Table 2:** Abundance (individuals per 100 g dry soil) of soil nematode genera in pumpkin and melon fields. PM, 4-year pumpkin monoculture; PR, 1-year pumpkin cropping; MM, 4-year melon monoculture; MR, 1-year melon cropping.

Genus	Trophic Group	*c*-*p* Value	PM	PR	MM	MR	Abbreviation
*Helicotylenchus*	Pp	3	33 ± 7	2 ± 0	4 ± 1	15 ± 3	*Helicoty*
*Pararotylenchus*	Pp	3	-	8 ± 1	-	-	*Pararoty*
*Pratylenchus*	Pp	3	14 ± 3	-	-	4 ± 1	*Pratylen*
*Paratylenchus*	Pp	3	-	21 ± 3	72 ± 17	21 ± 4	*Paratyle*
*Rotylenchus*	Pp	3	45 ± 9	8 ± 1	6 ± 1	4 ± 1	*Rotylenc*
*Aglenchus*	Pp	2	-	27 ± 4	-	-	*Aglenchu*
*Boleodorus*	Pp	2	8 ± 2	-	11 ± 2	23 ± 4	*Boleodor*
*Juveniles of Hoplolaimidae*	Pp	3	33 ± 7	27 ± 4	23 ± 5	13 ± 2	*Hoplolai*
*Alaimus*	Ba	4	-	6 ± 1	-	15 ± 3	*Alaimus*
*Acrobeles*	Ba	2	10 ± 2	27 ± 4	11 ± 2	8 ± 2	*Acrobele*
*Anaplectus*	Ba	2	29 ± 6	10 ± 2	32 ± 7	2 ± 0	*Anaplect*
*Acrobeloides*	Ba	2	70 ± 14	95 ± 15	86 ± 20	98 ± 19	*Acrobelo*
*Cephalobus*	Ba	2	29 ± 6	35 ± 5	-	17 ± 3	*Cephalob*
*Cervidellus*	Ba	2	-	8 ± 1	-	-	*Cervidel*
*Eucephalobus*	Ba	2	29 ± 6	143 ± 22	25 ± 6	48 ± 9	*Eucephal*
*Plectus*	Ba	2	33 ± 7	-	-	4 ± 1	*Plectus*
*Mesorhabditis*	Ba	1	-	31 ± 5	13 ± 3	44 ± 9	*Mesorhab*
*Protorhabditis*	Ba	1	8 ± 2	37 ± 6	-	46 ± 9	*Protorha*
*Rhabditidae*	Ba	1	-	4 ± 1	11 ± 2	8 ± 2	*Rhabditi*
*Ditylenchus*	Fu	2	16 ± 3	8 ± 1	21 ± 5	2 ± 0	*Ditylenc*
*Aphelenchoides*	Fu	2	-	25 ± 4	23 ± 5	19 ± 4	*Apheleno*
*Aphelenchus*	Fu	2	60 ± 12	39 ± 6	38 ± 9	40 ± 8	*Aphelenc*
*Filenchus*	Fu	2	41 ± 8	33 ± 5	23 ± 5	19 ± 4	*Filenchu*
*Tylencholaimellus*	Fu	4	-	-	-	13 ± 2	*Tylencho*
*Aporcelaimus*	OP	5	-	4 ± 1	-	4 ± 1	*Aporcela*
*Mononchus*	OP	4	-	-	4 ± 1	2 ± 0	*Mononchu*
*Eudorylaimus*	OP	4	25 ± 5	2 ± 0	17 ± 4	31 ± 6	*Eudoryla*
*Longidorella*	OP	4	6 ± 1	10 ± 2	4 ± 1	4 ± 1	*Longidor*
*Microdorylaimus*	OP	4	2 ± 0	33 ± 5	-	8 ± 2	*Microdor*

**Table 3:** Correlation of abundance, ecological indices, and metabolic footprint of soil nematodes with soil properties. PP, Ba, Fu, OP, and To indicate the abundance of plant parasites, bacterivores, fungivores, omnivores/predators, and total soil nematodes, respectively. H′, Shannon–Wiener index; PPI, maturity indices of plant-parasitic nematodes; MI, maturity indices of free-living nematodes. EI, enrichment index; SI, structure index; CI, channel index. PPF, BaF, FuF, and OpF indicate the metabolic footprints of plant parasites, bacterivores, fungivores, and omnivores/predators, respectively. Correlation coefficients labeled with ‘*’ and ‘**’ are significantly different at the levels of *p* < 0.05 and *p* < 0.01, respectively.

	SOC	Total N	Total P	Available P	Alkeline-N	pH
PP	−0.72 **	−0.67 *	−0.77 **	−0.74 **	−0.7 *	−0.65 *
Ba	0.39	0.63 *	0.76 **	0.52	0.71 **	0.73 **
Fu	−0.44	−0.30	−0.38	−0.43	−0.33	−0.31
OP	0.58	0.57	0.78 **	0.47	0.75 **	0.81 **
To	0.18	0.46	0.58 *	0.29	0.56	0.60 *
H′	0.49	0.67 *	0.71 **	0.72 **	0.72 **	0.58 *
PPI	−0.68 *	−0.77 *	−0.80 **	−0.66 *	−0.82 **	−0.80 **
MI	−0.09	0.11	0.08	−0.18	0.06	0.27
EI	0.79 **	0.52	0.69 *	0.41	0.71 **	0.70 *
SI	0.81 **	0.52	0.74 **	0.49	0.75 **	0.76 **
CI	−0.87 **	−0.67 *	−0.93 **	−0.92 **	−0.95 **	−0.74 **
PPF	−0.48	−0.36	−0.52	−0.87 **	−0.50	−0.20
BaF	0.25	0.50	0.64 *	0.42	0.58	0.58 *
FuF	−0.75	−0.56	−0.80	−0.75	−0.79	−0.68 *
OpF	0.63 *	0.28	0.49	0.23	0.46	0.54

## Data Availability

Not applicable.

## Supplementary Materials

The following are available online at https://www.mdpi.com/article/10.3390/life12010102/s1, Table S1: Soil properties in pumpkin and melon fields; Figure S1: Yields of pumpkin and melon.
