# Supplementary material for: Response of Soil Nematode Community Structure and Function to Monocultures of Pumpkin and Melon
Source: Life (Basel). 2022 Jan 12;12(1):102. doi: 10.3390/life12010102 (PMC8779376; doi:10.3390/life12010102)
Supplement: Supplementary file 1 [file life-12-00102-s001.zip › life-1517654-supplementary.pdf]

Supplementary Table S1. Soil properties in pumpkin and melon fields.

	SOC (g/kg)	pH	Total N (g/kg)	Total P (mg/kg)	Available P (mg/kg)	Alkeline-N (mg/kg)
PM	32.10 ± 0.03b	6.18 ± 0.03b	2.29 ± 0.03b	836.40 ± 0.33b	227.20 ± 1.80b	156.87 ± 6.22b
PR	35.54 ± 0.08a	6.47 ± 0.04a	2.88 ± 0.02a	918.77 ± 1.56a	297.80 ± 1.33a	198.50 ± 1.87a
MM	33.78 ± 0.02b	5.99 ± 0.02b	2.21 ± 0.42	845.67 ± 0.78b	281.77 ± 0.89b	164.43 ± 1.22b
MR	40.80 ± 0.67a	6.56 ± 0.04a	2.89 ± 0.02	931.33 ± 0.58a	301.57 ± 2.02a	208.87 ± 0.18a

PM, 4-year pumpkin monoculture; PR, 1-year pumpkin cropping; MM, 4-year melon monoculture; and MR, 1-year melon cropping. Lines with the same or no lower-case letters are not significantly different ($p < 0.05$) between PM and PR or MM and MR.

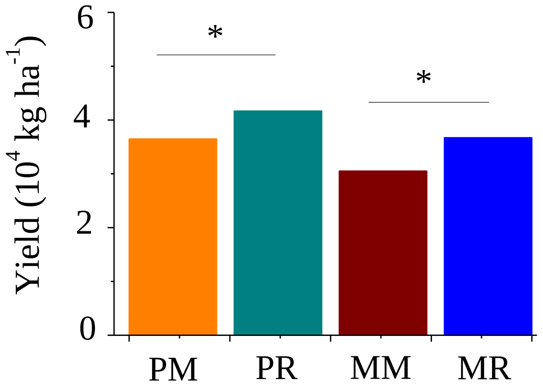

Supplementary Figure S1. Yields of pumpkin and melon. PM, 4-year pumpkin monoculture; PR, 1-year pumpkin cropping; MM, 4-year melon monoculture; MR, 1-year melon cropping. "*" indicates significant difference at $p < 0.05$ level.
